# The translation and psychometric assessment of the SCOFF eating disorder screening questionnaire: the Persian version

**DOI:** 10.1186/s40337-022-00564-3

**Published:** 2022-03-16

**Authors:** Shahin Bazzazian, Giti Ozgoli, Nourossadat Kariman, Malihe Nasiri, Tahereh Mokhtaryan-Gilani, Maryam Hajiesmaello

**Affiliations:** 1 Student Research Committee, Department of Midwifery and Reproductive Health, School of Nursing and Midwifery, Shahid Beheshti University of Medical Sciences, Tehran, Iran; 2 Midwifery and Reproductive Health Research Center, Department of Midwifery and Reproductive Health, School of Nursing and Midwifery, Shahid Beheshti University of Medical Sciences, Tehran, Iran; 3 Department of Basic Sciences, School of Nursing and Midwifery, Shahid Beheshti University of Medical Sciences, Tehran, IR Iran; 4 Department of Midwifery, Urmia Branch, Islamic Azad University, Urmia, Iran

**Keywords:** Psychometrics, Feeding and eating disorders, Surveys and questionnaires, Translations, Iran

## Abstract

**Background:**

Eating Disorders (EDs) are defined by abnormal eating habits. The SCOFF (Sick-Control-One stone-Fat-Food) is a simple screening questionnaire for EDs. This study was conducted to translate and evaluate the psychometric properties of the SCOFF questionnaire in Iranian university students.

**Methods:**

A total of 310 Iranian students of the Shahid Beheshti University of Medical Sciences completed a test battery of questionnaires including the well-known screener of eating disorder symptoms, the SCOFF. All measures were presented to the participants in Persian. The 5-item SCOFF questionnaire was translated to Persian using the forward–backward method. The face, content, criterion, and construct validity of the Persian version of the SCOFF were assessed. The validity and reliability of the Persian version of the SCOFF was assessed and factor analysis was conducted.

**Results:**

All five items of the translated questionnaire were approved after face validity. Content validity ratio was 0.73 (range 0.66–0.83) and content validity index was 0.96 (range 0.91–1), so all items were approved. Exploratory factor analysis revealed a 2-factor structure, which explained 52.47% of the variance. Confirmatory factor analysis showed a very good goodness-of-fit for the 2-factor model. 2-factor and 1-factor models indicate a very good goodness-of-fit in females and adequate goodness-of-fit in males. Criterion validity showed an acceptable correlation between the SCOFF and the EDE-Q. Reliability was acceptable based on the stability [ICC = 0.905(95% CI .760–.962 p < 0.001)] and the internal consistency (KR20 = 0.4).

**Conclusion:**

Appropriate psychometric properties of the 5-item Persian version of the SCOFF (both models) were confirmed, suggesting its use as a valid questionnaire in EDs screening.

**Supplementary Information:**

The online version contains supplementary material available at 10.1186/s40337-022-00564-3.

## Plain English summary

Eating Disorders (EDs) are defined by abnormal eating habits that affect a person's health. Eating disorders are common in Iranian young people. Early recognition of EDs can reduce its physical, psychiatric, psychosocial outcomes, improve the prognosis, and minimize the impact on patients and their families. However, timely recognition of EDs is difficult because of the variety of signs and presentations. The SCOFF (Sick-Control-One stone-Fat-Food) is a valuable screening questionnaire for rapid identification of people at risk for EDs. This study was conducted to translate and evaluate the psychometric properties of the SCOFF questionnaire in Iranian university students. The results of our study suggest that the 5-item Persian version of the SCOFF is a valid questionnaire in EDs screening.

**Supplementary Information:**

The online version contains supplementary material available at 10.1186/s40337-022-00564-3.

## Background

Eating Disorders (EDs) are defined by abnormal eating habits that affect a person's health [1]. The prevalence (ranges) of anorexia nervosa (AN), bulimia nervosa (BN), and binge eating disorder (BED) was reported 2.8% (0–4.8%),1.5% (0–8.4%), and 2.3% (0–9.8%) in women and 0.3% (0–0.4%), 0.1% (0–1.3%), and 0.3% (0–0.5%) in men respectively [2]. The results of a systematic review suggest that AN, BN, and BED prevalence have increased based on DSM-5 criteria [3]. The prevalence of EDs in young women is more than in men [4]. Shape concern, regular dietary restraint, and objective binge eating episodes were common in Iranian undergraduate women who participated in a study, in a way that 13.2% of them experienced shape concern, and 5% reported regular dietary restraint and objective binge eating episodes [5]. According to a study, eating disorder behaviors (EDBs) are common in Iranian college students, and the occurrence of most EDBs have no differences by gender. Binge eating was the most common EDB in this study (24.7% in men and 27.5% in women) [6]. Based on diagnostic criteria of the eating attitudes test (EAT-26), 24.2 percent of surveyed middle school students were at risk for EDs in northwestern Iran[7]. Given the high prevalence rates of EDs identified in Iranian samples, it is important that reliable screening tools for detection of possible EDs are available in Persian language for use with Iranian patients. Early recognition of EDs can reduce its physical, psychiatric, psychosocial outcomes [8], improve the prognosis [9], and thereby minimize the impact on patients and their families [10]. However, timely recognition of EDs is difficult because of the variety of the signs and presentations [11].

One of the obstacles to early EDs diagnosis is the lack of short screening tools in Iran. Existing diagnostic tools such as EDI [12] or BITE [13] take a long time to complete and are not easy to use [9]. In the UK, Morgan et al. (1999) designed a questionnaire called SCOFF (Sick-Control-One stone-Fat-Food) for a short, and simple screening questionnaire for EDs, which has five questions with good predictive validity and has been widely used [9].

The SCOFF questionnaire has been translated and evaluated in several countries, including Sweden [14], Mexico [15], Spain [16], Lebanon [17], China [18], France [19], Italy [20], and some other countries. Factor analyses revealed one-factor in some versions of the SCOFF, such as Arabic [17], Germany [21], and Italian [20], and two-factor in some others such as Finnish [22], and Catalan [23] versions of it. In the Swedish version of the SCOFF, the one-factor model had a good fit for girls and a very good fit for boys [14]. In the Mexican version, the one-factor model was favorable only for females; However, the 2-factor model was more favorable for both females and males [15]. A valuable screening questionnaire has been introduced that allows for the rapid and accurate identification of people at risk for EDs [24].

Given the importance of eating disorders among young people whose health is essential to achieve health promotion goals, it is necessary to validate an important tool that can help diagnose these disorders quickly and accurately. The present study was conducted to translate and determine the psychometric properties of the SCOFF questionnaire in Iran for the first time.

## Methods

The present study was conducted to translate and assess the psychometric properties of the Persian version of the SCOFF questionnaire.

**Fig. 1:**
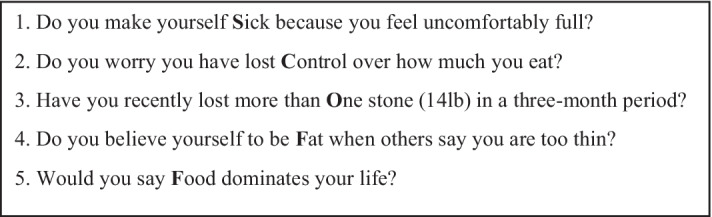
SCOFF questionnaire

The SCOFF questionnaire includes 5 yes/ no questions, scored from 0 to 5 according to the number of positive answers (Fig. 1). The questions of The SCOFF were developed using focus groups of patients with eating disorders and experts in eating disorders. Acceptability of the questions and the term “SCOFF” were reported by designers of the main version of the SCOFF. A positive SCOFF is defined by at least 2 positive answers [9].

The main version developers reported 100% sensitivity for anorexia nervosa and bulimia and a false-positive rate of 12.5%, which is considered an acceptable trade-off for high sensitivity [9]. The main version of the SCOFF was introduced in 5 items [9] without mention of its factor analyses, based on our extensive search. Some studies presented a one-factor model of the SCOFF [17, 21], and some others presented a two-factor model of it [22, 23]. According to the literature, the SCOFF may yield two factors reflecting different aspects of EDs, which consisted of Loss of Control Over Food [items 2, 4, and 5] and Purging Behaviors [items 1 and 3] [23]. In the current study, World Health Organization (WHO) protocol was used for psychometric and translation of this questionnaire [25].

### Translation procedure

In the first step, written permission for translation was obtained from the SCOFF designer. Then, the standard Forward–Backward translation method was used [25].

According to this method, the questionnaire was translated into Persian by two expert Persian translators separately, who were fluent in English, one of them was familiar with medical terms. A single Persian version of this questionnaire was prepared after evaluating and integrating the two translations by a panel of experts, some of the authors of this article, and the two translators.

In the next step, it was translated back into English by two other translators who were unfamiliar with the original questionnaire. Then, these two translations were reviewed and combined. The latest English version was sent to the SCOFF designer, and confirmation of it was received. (Translated Persian version of the SCOFF questionnaire was presented as Additional file 1).

The two main characteristics of tool psychometrics are validity and reliability [26]. To assess the psychometric properties of the Persian version of the SCOFF questionnaire, Face validity (quantitative and qualitative), content validity (quantitative and qualitative), criterion validity, construct validity [(Exploratory factor analysis (EFA) and confirmatory factor analysis (CFA)], and reliability of the questionnaire were examined. The statistical analysis was done by SPSS version 20. EQS software version 6.1 was used for factor analysis. Descriptive statistics (mean, standard deviation [SD], number, and percentage) were calculated for demographic variables.

### Face validity

Face validity is the degree to which a measure appears to be appropriate for collecting specific information, especially in the judgment of respondents [26]. In this study Face validity (qualitative and quantitative) of the SCOFF questionnaire was evaluated. In the qualitative face validity, 13 students from the target group were asked to comment on the relevancy, level of difficulty, and ambiguity of the questionnaire items. Then proper modifications were applied to the items according to received comments. In the quantitative face validity, each items’ impact score was determined. Items with an impact score ≥ 1.5 are considered appropriate and retained for further analysis [27].

### Content validity

The purpose of the content validity is to ensure the ability of the tool to measure the concept that it claims to measure [28]. In the qualitative content validity, 12 experts (psychologist, nutrition, and reproductive health experts) assessed the grammar, wording, and proper scoring of the SCOFF.

For the quantitative content validity, both the Content validity ratio (CVR) and content validity index (CVI) were measured. For this purpose, 12 experts in nutrition, reproductive health, and psychology (half of them was the same experts in qualitative content validity) were asked to score the essentiality of the SCOFF questionnaire items into three categories, including “Essential”, “Useful but not essential”, and “Not essential” from 1 to 3 respectively, based on Lawshe’s method. CVR was calculated in the following formula: CVR = (ne – (N/2)) / (N/2). In this formula, ne is the number of experts who rate an item as ‘Essential’ and N is the total number of experts. The calculated CVR was compared with the minimum acceptable CVR according to Lawshe’s table. Items with CVR more than that stated in the table for the given number of experts were considered necessary [29].

To measure CVI, we asked the same 12 experts to rate the relevance of the SCOFF questionnaire items on a four-point Likert scale from 1 to 4. CVI was calculated in the following way: Dividing the number of experts who had rated 3 or 4 for an item by the total number of experts. The Content validity index score above 0.79 is considered appropriate [30].

In the next step, two schools of Shahid Beheshti University of Medical Sciences were selected by simple random sampling. Participants were selected by convenience method. Being a student and a willingness to participate in research were considered as the inclusion criteria. The exclusion criterion was a lack of interest in participate in research. Participants answered the SCOFF questionnaire by the self-report method.

There are different views on the number of participants required to determine to construct validity in factor analysis. The recommended number of participants samples for analysis is at least 3–10 participants’ samples per tool item [26]. An additional rule of thumb with regard to sample size for factor analysis states that participant size 50 is very poor, 100 is poor, 200 is fair, 300 is good, 500 is very good, and 1000 is excellent [31]. Therefore, in the present study a sample size of 310 participants was considered most appropriate for the purpose of conducting factor analyses.

### Construct validity

To evaluate the construct validity in EFA, a principal components factor analysis with direct oblimin rotation was conducted on half of the total number of participants, selected at random (N = 153). Factor loading more than 0.4 considered acceptable for the presence of each item in a factor, using the following formula: CV = 5.152 ÷ √ (n − 2), in which CV = the number of extractable factors, and n = the sample size [32].

Confirmatory factor analysis (CFA) was performed for other half of participants (N = 154) and also by gender for two models (2-factors and 1-factor) using EQS 6.1 software. Root Mean Square Error of Approximation (RMSEA), Standardized Root Mean Square Residual (SRMR), Comparative Fit Index (CFI), Goodness of Fit Index (GFI), and Adjusted Goodness of Fit Index (AGFI) was used for assessment of the Model fit. Various cutoffs have been proposed by experts for fitness indicators. For example, a value equal to or less than 0.05 for RMSEA, a value equal to or greater than 0.96 for CFI, a value equal to or less than 0.07 for SRMR, it indicates that the model is adequately fitted [33]. On the other hand, it is suggested that if the CFI, GFI, and AGFI are greater than 0.9 and RMSEA and SRMR Less than 0.05 indicates a very good fit and less than 0.1 indicates a good fit [34].

### Criterion validity

Criterion validity could be determined by comparing the results of one instrument to results from another one intended to measure the same criterion [26]. In this study, the criterion validity of the translated SCOFF questionnaire was determined by the Persian version of Eating Disorder Examination Questionnaire (EDE-Q) [5] using the concurrent method. Pearson correlation was used for criterion validity (EDE-Q / SCOFF).

### Reliability

The reliability of the questionnaire was assessed by the stability and the internal consistency of the questionnaire. The stability of the questionnaire was assessed by the test–retest method using the intra-class correlation coefficient (ICC). Thus, 20 students were asked to answer the questions of the Persian version of the SCOFF questionnaire twice in two weeks. This number was not included in the next sampling. The internal consistency of the questionnaire was assessed using Kuder-Richardson (KR20).

## Results

Out of 310 students participating in the research, 3 withdrew and finally 307 questionnaires were completed. There wasn't any missing value on any items of questionnaire. According to the results, the participants' mean (SD) of age was 21.9 (4.27) years (range 17–49). Table 1 shows the frequency distribution and demographic characteristics of the participants. Considering at least two positive answers as a positive SCOFF [9], approximately 29% of students were at risk in the present study.

**Table 1:** Frequency distribution of demographic characteristics

Variable	Frequency n (%)
*Gender*	
Female	207 (67.4%)
Male	100 (32.6%)
*Degree*	
Bachelor	231 (75.2%)
Masters	24 (7.8%)
PhD	33 (10.7%)
Missing	19 (6.3%)
*Field of study*	
Nursing	198 (64.5%)
Operating room technician	22 (7.2%)
Reproductive health	10 (3.3%)
Midwifery	44 (14.3%)
Pharmacology	19 (6.2%)
Missing	14 (4.5%)
*Resident address*	
Student dormitory	116 (37.9%)
Pension	8 (2.6%)
Parent home	149 (48.5%)
Relatives/ friends home	17 (5.5%)
Missing	17 (5.5%)
*Marital status*	
Unmarried	264 (86%)
Married	29 (9.4%)
Missing	14 (4.6%)

All of the questionnaire items were translated in a simple, clear, and relevant way. After qualitative face validity, all 5 items of the questionnaire were approved in terms of the level of difficulty, relevancy and, ambiguity. In face validity, the Impact Score of all items was more than 1.5 (in the range of 3.19–4.69), so all items were retained for further analysis.

In the present study, the number and content of the questionnaire items did not change after qualitative content validity. The CVR was calculated 0.73 for the entire questionnaire and ranged from 0.66 to 0.83 for each item. Considering that the number of experts was 12, items with a value of > 0.56 were acceptable according to Lawshe’s table [29]. Therefore, all of the questionnaire items were retained. Also, the CVI was calculated at 0.96 for the entire questionnaire and ranged from 0.91 to 1 for each item. Since the content validity index score is higher than 0.79 [30], thus, all the questionnaire items consider appropriate in terms of relevance.

To determine the exploratory factor analysis of the questionnaire 153 eligible samples (more than 30 samples for each item = the most ideal sample size) [25, 26] were randomly selected. Principal components factor analysis with direct oblimin rotation revealed a two-factor structure with eigenvalues of > 1, which explained 52.47% of variance [Kaiser–Meyer–Olkin (KMO) = 0.541, Barlett test of sphericity P < 0.001] (Table 2). Factor 1[Loss of Control Over Food] included items 2, 4, and 5. Factor 2[Purging Behaviors] included items 1, 3.

**Table 2:** Exploratory factors extracted from items of the SCOFF (N = 153)

Factor	Q_n_.Item	Factor loading before rotation		Factor loading after rotation**		h^2^*	%Variance	Eigenvalue
Loss of Control Over Food	2. Do you worry you have lost **C**ontrol over how much you eat?	0.617	–	0.651	–	0.432	29.715	1.486
	4. Do you believe yourself to be **F**at when others say you are too thin?	0.752	–	0.772	–	0.594		
	5. Would you say **F**ood dominates your life?	0.550	–	0.514	–	0.320		
Purging Behaviors	1. Do you make yourself **S**ick because you feel uncomfortably full?	0.475	0.600	–	0.661	0.586	22.753	1.138
	3. Have you recently lost more than **O**ne stone (14 lb) in a three-month period?	–	0.825	–	0.804	0.692		

*Communality

**Direct oblimin rotation

Table 2 shows the factor loading of the SCOFF questionnaire before and after rotation. As shown in Table 2 all factor loadings were appropriate after rotation and there was no cross loading. In other words, items 2, 4, and 5 were loaded only in factor one and items 3 and 1 in factor two. The smallest factor loading belonged to question 5 with a value of 0.514 from the “Loss of Control Over Food” component, but the largest factor loading was related to question 3 with a value of 0.804 from the “Purging Behaviors” component.

**Fig. 2:**
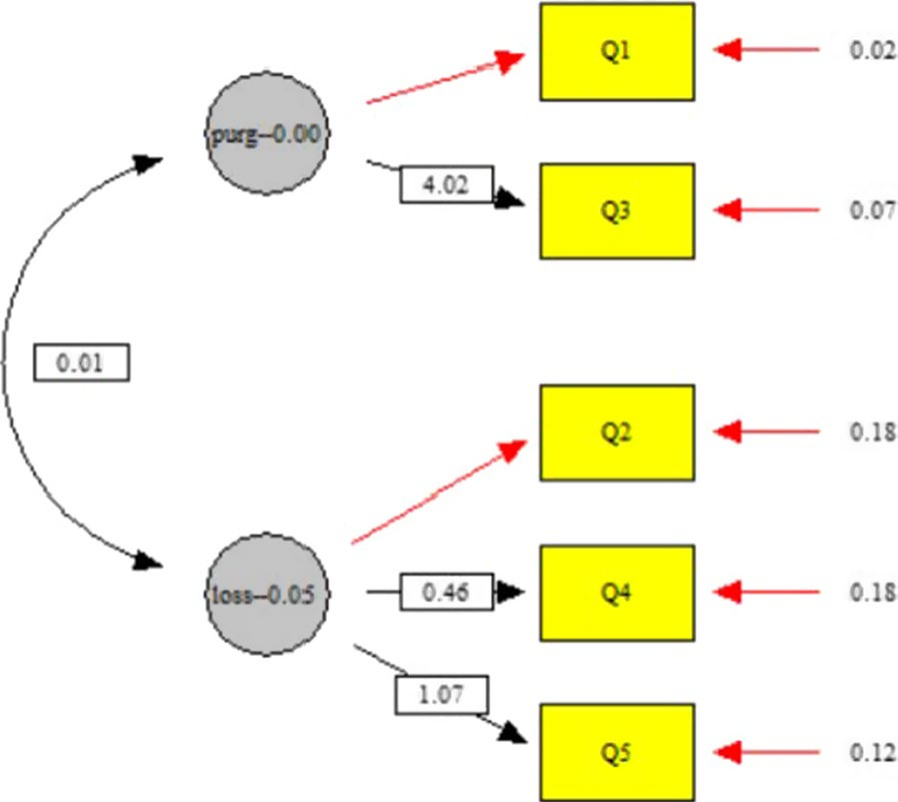
2-factor model of the SCOFF questionnaire

In the construct validity stage, confirmatory factor analysis was used for the 2-factor model. In this model, questions 1, and 3 of the questionnaire are in the field of purging behavior, and questions 2, 4, 5 are in the field of loss of control over food solution [15] (Fig. 2).

According to the output of EQS, the 2-factor model had quite good fit indices. Table 3 presents the fit of a 2-factor model of the SCOFF. As shown in Table 3, in this model, GFI, AGFI, and CFI are greater than 0.9, and SRMR is less than 0.05, which indicates a very good fit and RMSEA is less than 0.1, which indicates a good fit. Also, according to the value of the Chi-square mean/degree of freedom (CMIN / DF) in this model, the quality of the model is confirmed.

**Table 3:** Fitting Indices of 2-factor Model of SCOFF questionnaire

Indices*Model (n = 154)	χ^2^	df	P value	CMIN/DF	RM SEA	AGFI	GFI	CFI	SRMR
2-factor Model	6.840	4	0.14	1.71	0.068	0.934	0.983	0.924	0.044

RMSEA: Root Mean Square Error of Approximation; SRMR: Standardized Root Mean Square Residual; GFI: Goodness of Fit Index; AGFI: Adjusted Goodness of Fit Index; χ^2^: Chi-square; df: degree of freedom; CMIN/DF: Chi-square mean/degree of freedom; CFI: Comparative Fit Index

*Acceptable values are as follows: > 0.9 for AGFI, GFI; < 0.09 for SRMR; < 0.08 for RMSEA; and < 3 for CMIN/DF

To evaluate the fitness of the questionnaire in females and males, a comparison of 2-factor and 1-factor models was performed in females and males separately. The results were obtained according to Tables 4 and 5:

**Table 4:** Comparison of 2-factor and 1-factor models in females

Indices* Model (n = 207)	χ^2^	df	P value	CMIN/DF	RMSEA	AGFI	GFI	CFI	SRMR
2 factors model	4.144	4	0.39	1.04	0.013	0.970	0.992	0.994	0.035
1 factor model	7.543	5	0.18	1.50	0.050	0.956	0.985	0.901	0.047

*Acceptable values are as follows: > 0.9 for AGFI, GFI, CFI; < 0.09 for SRMR; < 0.08 for RMSEA; and < 3 for CMIN/DF

**Table 5:** Comparison of 2-factor and 1-factor models in males

Indices* Model (n = 100)	χ^2^	df	P value	CMIN/DF	GFI	SRMR	RMSEA	AGFI	CFI
2 factors model	10.419	4	0.03	2.60	0.962	0.067	0.127	0.859	0.764
1 factor model	11.619	5	0.04	2.32	0.961	0.070	0.116	0.883	0.757

SRMR: Standardized Root Mean Square Residual; GFI: Goodness of Fit Index; χ2: Chi-square; df: degree of freedom; CMIN/DF: Chi-square mean/degree of freedom; AGFI: Adjusted Goodness of Fit Index; CFI: Comparative Fit Index; RMSEA: Root Mean Square Error of Approximation

*Acceptable values are as follows: > 0.9 for GFI; < 0.09 for SRMR; and < 3 for CMIN/DF

RMSEA: Root Mean Square Error of Approximation; SRMR: Standardized Root Mean Square Residual; GFI: Goodness of Fit Index; AGFI: Adjusted Goodness of Fit Index; CFI: Comparative Fit Index; χ2: Chi-square; df: degree of freedom; CMIN/DF: Chi-square mean/degree of freedom.

As shown in Table 4, in both the two-factor and one-factor models of females, CFI, GFI, and AGFI greater than 0.9 and RMSEA and SRMR are less than 0.05, which indicates a very good fit. Also, according to the value of CMIN / DF in both models, the quality of the model is confirmed. Therefore, both two-factor and one-factor models in females based on RMSEA, AGFI, SRMR, GFI, CMIN / DF, CFI fit indices have a very good data-model fit.

As shown in Table 5, in both two-factor and one-factor males models, GFI is greater than 0.96 and acceptable, and SRMR in both models is equal to or less than 0.07 indicating adequate fit of the model. Also, according to the value of CMIN / DF in both models, the quality of the model is confirmed. Therefore, both two-factor and one-factor models of males have adequate data-model fit based on SRMR, GFI, CMIN / DF fit indices.

Criterion validity using the concurrent method was performed to determine the correlation between SCOFF and EDE-Q questionnaires. The results showed an acceptable correlation between SCOFF and EDE-Q for females, males, and the total of participants (Table 6):

**Table 6:** Correlation between SCOFF and EDE-Q

	Scoff total					
	Females N = 207	P value	Males N = 100	P value	Total N = 307	P value
	r	P value	r	P value	r	P value
EDE-Q total	0.492**	< 0.001	0.529**	< 0.001	0.496**	< 0.001

**Correlation is significant at the 0.01 level (2-tailed)

The reliability of the questionnaire was assessed by the internal consistency method. The internal consistency of the questionnaire using the Kuder-Richardson test (KR20 = 0.4), which is considered acceptable for screening questionnaires [35]. The stability of the questionnaire was assessed by the test–retest. So, the intra-class correlation coefficient was acceptable [ICC = 0.905 (95% CI 0.760–0.962, p < 0.001)].

## Discussion

The present study was conducted to examine the psychometric properties of the Persian version of the SCOFF questionnaire for the first time in the Iranian samples. 5-items of the questionnaire were translated to Persian using the forward–backward method. The Persian version of the questionnaire showed acceptable face and content validity.

In the present study, EFA revealed a two-factor model of the SCOFF with acceptable factor loadings of items (0.514–0.804). The two extracted factors together explained 52.47% of the variance. These findings are similar to the results of the Catalan version. In the two-factor model of that version, factor loadings of the items were 0.591, 0.877 for questions 1, 3 from factor 2(Purging Behaviors) and 0.844, 0.812, and 0.462 for questions 2, 4, and 5 from factor 1(Loss of Control Over Food) respectively, which explained 55.57% of the variance [23].

On the other hand, in the Arabic version of the SCOFF, EFA revealed one factor that explained 31% of the variance. Factor loadings were also between 0.30 and 0.75 [17]. The participants of the Arabic version were selected from primary healthcare centers, and the male gender was exclusion criteria, which may explain the differences between its results and the present study.

In the present study, the largest factor loading was related to question 3. However, in the Italian version of the SCOFF, factor loadings were between 0.33 and 0.66 (Q1, 0.57; Q2, 0.63; Q3, 0.33; Q4, 0.40; and Q5, 0.66), and question 3 had the smallest factor loading [20]. Differences in participants may be able to explain this, considering that some of them were ED patients in the Italian study. Also, the authors of the Italian version of the SCOFF explained the low loading of question 3 by its objective content (loss of weight), which maybe make it different from others with subjective content [20]. These reasons may explain the differences between our finding of this question and the Italian version of the SCOFF.

In the confirmatory factor analysis of the current study, EQS output indicated very good fit indices (RMSEA, AGFI, GFI, CFI, SRMR, CMIN / DF) for the 2-factor model, which confirms the questionnaire in the Iranian sample. Also, the CFA of both models (2-factor and 1-factor) indicated a suitable fit for females and males separately. These results are similar to the Swedish study, in which the one-factor model had a good fit for girls and a very good fit for boys [14]. It is also similar to the Italian study for girls [20].

On the other hand, the CFA results of the Mexican study indicated that the 1-factor model was favorable only for females, and the 2-factor model was more favorable for both females and males [15]. These results are different from the present study, in which the 1-factor model showed a good fit for males. This difference can probably be explained by the lower average age of participants in the Mexican study (18.1 years in the Mexican study versus 21.9 years in the current study) and the larger number of participants in the Mexican study.

In the present study, the acceptable correlation coefficient between the two questionnaires SCOFF/EDE-Q indicated the effectiveness of the SCOFF questionnaire compared to the EDE-Q questionnaire. A Swedish study indicated a significant correlation between the SCOFF and the EDE-Q in male and female samples [14], which is similar to the results of the present study. According to a Chinese study, the SCOFF scores were significantly correlated with the total scores of the EDE-Q and both the EDE-Q and the SCOFF showed significantly higher scores in participants with the potential of having eating disorders [18]. These results are also similar to the results of the current study.

According to the current study results, the ICC was above 0.9, which indicates the stability of this questionnaire. Internal consistency (KR20 = 0.4) was similar to the results of the Swedish (KR20 = 0.48) [14] and the Mexican study (KR20 = 0.49 for females and KR20 = 0.59 for males) [15].

The strengths of the present study are conducting the criterion validity using the concurrent method and determining factor analysis by gender that provides valuable information.

One of the limitations of the current study is that the samples are only from university students. Sampling from clinics, health centers, or populations with a high risk of eating disorders may cause different results. Another limitation is that most samples are young (age mean = 21.9). Therefore, sampling in mentioned settings with more variety in age groups is suggested.

## Conclusion

Results of the current study indicated that the Persian version of the SCOFF questionnaire, has acceptable psychometric properties and it can be used as a valid questionnaire for screening of eating disorders in Iran. The simple 5-question makes it easier to use and does not require much time and can help to rapid and timely diagnosis of eating disorders.

Therefore, it is recommended that the SCOFF questionnaire be included in the medical examinations and be used to screening eating disorders in the Iranian community to prevent the complications of eating disorders by timely diagnosis and early intervention.

## Supplementary Information

**Additional file 1**. The Persian version of the SCOFF questionnaire.

## Data Availability

The datasets used during the present study can be accessed from the corresponding author on reasonable request.

## Abbreviations

SCOFF: Sick-Control-One stone-Fat-Food
EDE-Q: Eating Disorder Examination Questionnaire
EDs: Eating Disorders
AN: Anorexia Nervosa
BN: Bulimia Nervosa
BED: Binge Eating Disorder
CFA: Confirmatory factor analysis
EFA: Exploratory factor analyses
CFI: Comparative Fit Index
CVI: Content validity index
CVR: Content validity ratio
ICC: Intraclass correlation coefficient
RMSEA: Root Mean Square Error of Approximation
SRMR: Standardized Root Mean Square Residual
GFI: Goodness of Fit Index
AGFI: Adjusted Goodness of Fit Index
KMO: Kaiser–meyer–olkin
CMIN/DF: Chi-square mean/degree of freedom
