# Supplementary material for: The translation and psychometric assessment of the SCOFF eating disorder screening questionnaire: the Persian version
Source: J Eat Disord. 2022 Mar 16;10:38. doi: 10.1186/s40337-022-00564-3 (PMC8925224; doi:10.1186/s40337-022-00564-3)
Supplement: Supplementary file 1 — Additional file 1. The Persian version of the SCOFF questionnaire. [file 40337_2022_564_MOESM1_ESM.docx]

**Supplementary material**

The Persian version of the SCOFF questionnaire:

1-آیا شما از شدت احساس ناخوشایند پر خوری، خودتان را عمدا مجبور به استفراغ می کنید؟ بلی خیر

2- آیا شما از این که کنترل میزان غذا خوردن خود را از دست داده اید ، نگران هستید؟ بلی خیر

3- آیا اخیراً کاهش وزنی به میزان 7-6 کیلو در عرض سه ماه داشته اید؟ بلی خیر

4- آیا شما به نظر خودتان چاق هستید درحالی که دیگران به شما می گویند خیلی لاغرید؟ بلی خیر

5- آیا به نظر شما غذا سایر جنبه های زندگی شما را تحت تسلط خود درآورده است؟ بلی خیر
